# COVID-19 social distancing: negative effects on people with Parkinson disease and their associations with confidence for self-management

**DOI:** 10.1186/s12883-021-02313-6

**Published:** 2021-07-20

**Authors:** Galit Yogev-Seligmann, Michal Kafri

**Affiliations:** 1 Department of Occupational Therapy, Faculty of Social Welfare and Health Sciences, University of Haifa, 3498838 Haifa, Israel; 2 Department of Physical Therapy, Faculty of Social Welfare and Health Sciences, University of Haifa, Haifa, Israel

**Keywords:** COVID-19, Parkinson disease, Self-management, Social distancing, Patient activation

## Abstract

**Background:**

The purpose of this study was to describe the effects of COVID-19 social distancing on the function, health, and well-being of people with Parkinson disease (PD), and test the association of these effects with patients’ activation levels, i.e., their skills and confidence in managing their health.

**Methods:**

Community-dwelling individuals with PD answered an anonymous web-based survey. Part 1 included 27 multiple-choice questions regarding changes in function, health, medical care, and well-being. Part 2 consisted of the Patient Activation Measure, which enquired about skills and confidence in managing one’s health.

**Results:**

Respondents (*N* = 142) reported decreases in various function (24.8%–37.3%), health (33.8%–43%), and well-being (26.1%–47.1%) domains. Rehabilitation ceased for 61.2%. Among those reporting a worsening of health, 67.8% associated this with the cessation of rehabilitative treatments or decrease in physical activity.

Patients’ activation levels were inversely correlated with increased assistance for activities of daily living, increased tiredness, worsening symptoms, and lack of support from family and friends.

**Conclusions:**

Social distancing had a major negative impact on the health and function of people with PD.

**Practical implications:**

Supporting people with PD skills and confidence in managing health may preserve their physical and mental health during this period of dramatic changes in life’s circumstances.

**Supplementary Information:**

The online version contains supplementary material available at 10.1186/s12883-021-02313-6.

## Introduction

People with chronic diseases, such as Parkinson disease (PD), who live in the community rely on community resources (e.g., outpatient clinics, exercise programs, and patient support groups) and social networks to maintain their health and well-being. For example, among people with PD, treatment by a neurologist and adherence to anti-parkinsonian medication has been positively associated with improved health-related outcomes, such as fewer medical complications and hospitalizations [1–5]. Moreover, social support, including visits from friends and family, and invitations to socialize with other people, has a prominent role in positive living with PD [6].

The lockdown and social distancing measures related to coronavirus disease 2019 (COVID-19) have posed enormous challenges to maintaining the health and well-being of the population, particularly those with chronic diseases [7–11]. A lockdown of more than eight weeks forced residents to stay in their homes, cease outdoor activities, and avoid socializing. This dramatic life change was accompanied by multiple consequences that impacted health and well-being, such as reduced spontaneous and structured physical activity, loss of support from family and friends, and reduced access to healthcare services.

A few papers have presented a range of possible effects of the COVID-19-related social distancing and disruption of normal routines in people with PD. These include decreased physical activity, worsening of disease symptoms, and increased stress and anxiety [8, 11, 12]. Researchers have also anticipated that people will have difficulty adapting to these new circumstances [12]. Cumulative evidence from empirical studies supports some of these implications. For instance, studies have found evidence for reduced physical activity, worsening of motor and non-motor symptoms, difficulty acquiring neurology consultations or medications, increased anxiety, sleep disturbances, and increased burden on family members or caregivers [13–18]. In addition, associations have also been observed between the psychological impact of the COVID-19 pandemic and patients’ background characteristics, such as disease severity, on reduced physical activity [14, 15]. Helmich and Bloem [12] have pointed out that the COVID-19 crisis provides an opportunity to identify the determinants of coping well with unanticipated life-changing circumstances. In this context, we were specifically interested in the possible contribution of patients' capacities for self-management, as these are modifiable factors related to their health and well-being. Self-management is considered a pivotal element in the management of chronic diseases [19], and accordingly, has become the focus of many healthcare policies [20, 21]. Driven by the task-model approach, which explains the process of adaptation to chronic illness [22], self-management encompasses three essential components: behavioral management, such as adapting one’s lifestyle to the changes brought about by the illness; medical management, such as attending medical appointments; and emotional management, such as processing emotions that arise from having a chronic illness. The ability to perform these tasks has been severely hindered due to the social distancing policies adopted under the COVID-19 pandemic.

The concept of patient activation emerged in accordance with the recognition that self-management behaviors play an important role in the long-term management of chronic diseases [23–25]. This concept describes people’s knowledge, skills, and confidence in managing their health. The patient activation measure is commonly used to describe a patient’s level of confidence and ability to self-manage effectively. Compared to people with low levels of activation, people with high levels of activation are more likely to adopt self-management behaviors such as maintaining physical activity [26]; thus, they are more likely to have better clinical outcomes [24, 25]. It is possible that activation levels not only interact with the maintenance of self-management behaviors, but also improve people’s capacity to effectively adjust their self-management behaviors to new circumstances, such as those imposed by the COVID-19 pandemic.

Within this context, the current survey had two major objectives. The first was to describe the effects of COVID-19 social distancing and lockdown on the function, health, and well-being of people with PD. The second was to test the association between the patients’ activation levels and these effects. We hypothesized that people who have stronger activation―a higher level of knowledge about their disease and its treatment, and are skilled and confident in managing their disease―would have adjusted better to the changes in life that may have occurred due to the COVID-19 pandemic. We expected the evidence in support of this hypothesis to be indicated by significant correlations between patients’ activation levels and changes in function, health, and well-being.

## Methods

### Sample

People with PD were invited to answer the survey if they were over 18 years old, lived in the community, could read and understand Hebrew, and had not been diagnosed with COVID-19 or hospitalized in the last three months. A sample size calculation indicated that a sample of at least 90 respondents would be sufficient to reach a value of r ≥ 0.35, with a statistical power of 0.8 and α = 0.01.

### Study design and procedure

A cross-sectional web-based survey was conducted. The invitation to participate in the survey was distributed via social media networks and through the Israeli Parkinson Association. The invitation included a link to the survey. The survey was anonymous, and participants were able to complete it on a smartphone or computer.

All study procedures were approved by the Ethics Committee of the University of Haifa, and answering the survey was considered as an agreement to participate. All methods were performed in accordance with the relevant guidelines and regulations. The survey was conducted using a Qualtrics survey platform and was open for recruitment from 10 May, 2020, for three weeks. The survey asked respondents to reflect on the COVID 19-related lockdown period that started in Israel in mid-March. During this period, Israeli residents were under a mandatory lockdown in which they were allowed to walk outside their homes no farther than 100 m, and to leave the house only for essential needs. Social distancing required older adults or people with comorbidities to avoid physical contact with anyone not living in the same house. Gradual lifting of restrictions began in mid-May.

### Survey development and structure

The complete survey is presented in the Appendix. The survey consisted of two parts.

Part 1 was used to identify the effects of the lockdown on health, function, medical care, and well-being. It included general demographic information and 27 multiple-choice questions regarding the following:

- Current functional status**:** independence in mobility and activities of daily living (ADL), falls (Q1, Q2, Q4, Q13), and changes in these domains due to the lockdown (Q3, Q5, Q14).
- Health during the lockdown**:** controlling symptoms related to other comorbidities (Q15), weight gain (Q18), change in disease symptoms (Q20), self-reported estimates of the reason for worsening of symptoms (Q21), and overall disease condition (Q27).
- Medical care**:** cancelation of rehabilitative treatments (Q19), adherence to medication regimen during the lockdown and reason for change in adherence (Q24, Q25), continuation of scheduled visits to the neurologist (Q26), and frequency and type of physical activity (Q22, Q23).
- Well-being during the lockdown**:** frequency of tiredness, depression, isolation, anxiety, and worry about the future (Q6–Q9, Q16), whether their frequency changed compared to the period before the lockdown (Q10, Q17), and lack of family and social support during the lockdown (Q11, Q12).

Part 2 evaluated the patient activation level and consisted of the Patient’s Activation Measure (PAM-13®, Insignia Health) [27]. The PAM-13 is a self-reported, validated, and licensed tool to measure a patient’s knowledge, skills, and confidence for self-management. The overall score captures the extent to which people feel engaged and confident in taking care of their health conditions. It consists of 13 statements rated on a four-point Likert scale providing the following options: strongly disagree, disagree, agree, and strongly agree. The PAM-13 score is transformed into a 0–100 continuous scale according to a licensed conversion table (Insignia Health) [28], with higher scores indicating stronger activation [27]. Based on their PAM-13 score, people are divided into four ordinal levels of activation. Level 1 represents patients who tend to be passive and feel overwhelmed managing their own health, while level 4 represents patients who have effectively adopted self-management behaviors.

PAM-13 has been reported to be a reliable and valid instrument for research on patients with neurological conditions [29]. We used a validated, licensed Hebrew version of the PAM-13 supplied by Insignia Health (https://www.insigniahealth.com/products/pam-survey), which holds the copyright to the questionnaire.

### Statistical analysis

Demographic data were presented as mean ± standard deviation or frequencies. Responses for functional status, health during the lockdown, medical care, and well-being, and changes in these domains due to COVID-19 were reported as frequencies. PAM-13 data were reported according to the PAM levels and entered into the statistical analysis as ordinal variables.

A one-tailed Spearman’s rank correlation coefficient was used to analyze the associations between PAM-13 levels and changes in health, function, and well-being.

Strength of associations was reported as *r* < 0.3, weak; *r* = 0.3–0.5, fair; *r* = 0.6–0.8, moderately strong; and *r* =  < 0.8, very strong [30]. To further explore differences between respondents with high and low activation levels in the domains associated with PAM-13, we placed respondents into low activation (PAM-13 levels 1 and 2) and high activation (PAM-13 levels 3 and 4) groups, and conducted Pearson's chi-square tests of independence. For the questions on changes in disease symptoms and changes in tiredness, we excluded respondents who reported an improvement in symptoms and reduction in tiredness, as these questions included only three responses each. For the question on lack of family support, we combined the responses “a little bit,” “sometimes,” “often,” and “all the time” to a single level that indicated a lack of family support. Accordingly, all analyses produced 2 × 2 contingency tables.

## Results

A total of 142 people with PD (59% men) responded to the survey. The mean age of the respondents was 70.6 ± 7.6 years and the mean disease duration was 10.6 ± 8.3 years. Most respondents (*n* = 129, 91%) lived with another person at home. The complete survey results are presented in the Appendix. Table 1 summarizes the respondents’ functional characteristics (questions 1, 2, and 4).

**Table 1:** Respondents’ functional characteristics according to survey questions 1, 2, and 4

Survey question	Response	Response rate (%)
**1. Independence in walking**	Walking independently	65.2%
	Using cane or walker	31.9%
	Using wheelchair	2.8%
**2. Need for physical assistance while walking**	Walk independently	71.4%
	Require another person's supervision	20.0%
	Require another person's physical support and help	8.6%
**4. Help from another person with activities of daily living (ADL)**	Independent in all daily functions	63.4%
	Require supervision in some or all of my daily functions	14.1%
	Require a little help in some or all of my daily functions	18.3%
	Require medium to great help in some or all of my daily functions	4.2%

### Effects of social distancing and the lockdown on function, health, medical care, and well-being

#### Effects on functional domains

**Fig. 1:**
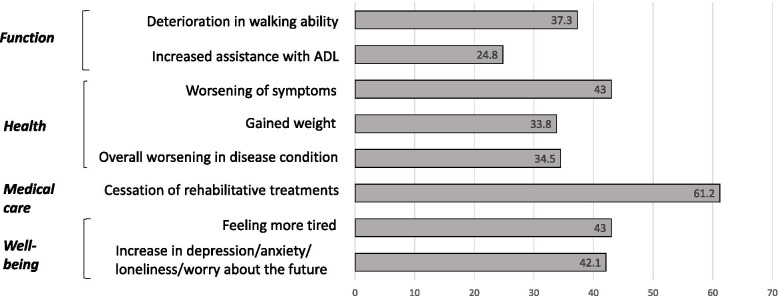
Effects of social distancing and lockdown on function, health, medical care and well-being. Bars represent the percentage of participants who reported a change in each of the domains during the lockdown period relative to the previous period

Thirty-seven percent of the respondents reported a deterioration in their walking ability, and 24.8% reported an increased need for assistance with ADL (Fig. 1). Although 18.3% reported falls during the lockdown, only 4.3% reported falling more often than before the lockdown.

#### Effects on health domains

Forty-three percent of the respondents reported a worsening of disease symptoms during the lockdown, while 33.8% reported weight gain. Most respondents (83.2%) were able to control other comorbidities; however, 34.5% of respondents reported an overall worsening of their disease condition (Fig. 1).

#### Effects on medical care

Sixty-nine percent of the respondents reported that their rehabilitative treatments ceased (Fig. 1). The most common reason reported for the cessation of rehabilitative therapy was cancelation by the service provider (61.2%), and fear to continue the treatment (7.2%). Among those reporting a worsening of symptoms, 67.8% associated this with the cessation of rehabilitative treatments or decrease in physical activity. Most respondents adhered to their medication regimen (97.2%), and only 9.2% reported that the lockdown affected their ability to meet with their neurologist. A total of 69.7% respondents reported that they exercised 3–5 times a week or every day. The type of exercise varied and included walking, stretching, yoga, stationary-biking, and exercising via the online video application Zoom or television.

#### Effects on well-being

Compared to the period before the lockdown, 43% of the respondents reported feeling more tired, and 42.1% reported increases in at least one of the following: depression, anxiety, loneliness, or worry about the future (Fig. 1).

Overall, 26.1% respondents reported a lack of spousal support during the lockdown, ranging from “a little” to “all the time”. Moreover, 47.1% reported a lack of support from family members or close friends, ranging from “a little” to “all the time.”

### Patient activation and its association with effects of social distancing and the lockdown

Twenty-five (17.6%) respondents were categorized as PAM-13 level 1, 21 (14.8%) as level 2, 50 (35.2%) as level 3, and 46 (32.4%) as level 4, which indicates the highest level of patient activation. PAM-13 level was inversely associated with increased assistance with ADL, worsening of symptoms, increased tiredness, and lack of support from family members and friends (Table 2).

**Table 2:** Association of patient activation measure level with effects of social distancing and the lockdown

Association with PAM-13 level (Spearman’s rank)	Variable
-0.23 (***p*** = **0.02)**	Increased assistance with ADL
-0.26 (***p*** < 0.001)	Worsening of disease symptoms
-0.31 (***p*** < 0.001)	Increased tiredness
-0.33 (***p*** < 0.001)	Lack of family members’ and friends’ support

*ADL* activities of daily living, *PAM* Patient Activation Measure

The Pearson’s chi-square test of independence showed a significant difference between people with high and low activation on the pattern of coping with social distancing and the lockdown (Table 3). People with activation levels 3 and 4 were more likely to maintain their levels of function, as indicated by changes in the assistance required for ADL. They were also less likely to report a worsening of disease symptoms, an increase in tiredness, or a lack of social support from friends.

**Table 3:** Proportion of low and high activation level respondents with reported change in measured PAM-13 domains

		Patient’s activation level		χ^2^(DF), p
		Low level of activation (PAM-13 levels 1 and 2, *n* = 46)	High level of activation (PAM-13 levels 3 and 4, *n* = 96)	
Increased assistance with ADL	No	58.7%	83.2%	χ^2^ (1) = 9.94, *p* = 0.002
	Yes	41.3%	16.8%	
Worsening of disease symptoms	No	40%	63.8%	χ^2^ (1) = 7.02, *p* = 0.008
	Yes	60%	36.2%	
Increased tiredness	No	33.3%	67%	χ^2^ (1) = 14.02 *P* < 0.001
	Yes	66.7%	33%	
Lack of family members’ and friends’ support	No	30.4%	63.5%	χ^2^ (1) = 13.68 *p* < 0.001
	Yes	69.6%	36.5%	

*ADL* activities of daily living, *PAM* Patient Activation Measure

## Discussion and conclusion

### Discussion

#### The effect of social distancing and the lockdown on function, health, healthcare, and well-being

The results of the current survey revealed that the social distancing and lockdown related to COVID-19 had a negative impact on people with PD. The most prominent was the decrease in rehabilitative care, experienced by approximately 60% of the participants. Similarly, Schirinzi et al. [14] reported that 78% of patients experienced a decrease in physical therapy or rehabilitation during the lockdown as compared to before. Our study results also indicated a reduction in well-being due to increased negative feelings (e.g., depression, loneliness, or worry) and tiredness, reported by approximately 40% of respondents. Moreover, approximately 40% of survey respondents reported changes in health, including a worsening of disease symptoms and weight gain. The extent of study respondents reporting a worsening in the physical and psychological domains of their health was similar to that found in other recent studies [13–18, 31]. In our study, more than two-thirds of the respondents associated canceled rehabilitative care with a worsening of disease symptoms. This subjective association between reduction in rehabilitative therapy and worsening of disease symptoms is provided credence by the objective finding that decreased physical activity was the main risk factor for worsening global health during the COVID-19 lockdown [14].

The negative effect on function, experienced by one-third to one-fourth of the respondents, was reflected by declines in the functional level of walking and an increased need for assistance with ADL. In contrast to physical rehabilitation, which was reduced dramatically, respondents were able to maintain other aspects of healthcare, including taking prescribed medications and attending neurology visits as scheduled. Together, these findings reflect the negative effects of the lockdown and social distancing on people with PD across all survey domains.

Paradoxically, fall status may have been preserved because people stayed at home and were not as prone to falls in the community. This idea is supported by several studies that have reported that 20–57% of the falls in people with PD occur outdoors [32–34].

The increase in negative feelings adds to the already high prevalence of mood disorders among people with PD [35]. This aspect is especially important because mood disorders are linked to negative health outcomes, decreases in daily function, and reduced quality of life [36–38]. Tiredness is an additional facet of the well-being domain. We used the term “tired” to capture some aspects of the multi-factorial concept of fatigue, which is defined as a feeling of exhaustion and absence of energy, distinguished from sadness or weakness [39]. Fatigue is very common among people with PD, and is reported by many as the most disabling symptom. It is common to classify mechanisms of fatigue development as physiological or psychological [40]. We speculate that the increase in reports of tiredness among the respondents reflects psychological mechanisms related to the overall increase in negative feelings. In addition, as people were forced to stay home, we think that the findings of increased tiredness were reflective of a general feeling of exhaustion rather than a reaction to physical effort. An increase in tiredness can also be partially associated with increased sleep disturbances [16, 17].

Approximately 47% of the respondents felt that they lacked social support from friends a little, sometimes, often, or all the time, as compared to approximately 53% who did not feel that they lacked social support. A recent cross-sectional study of people with PD [6] found that social support had the strongest influence on positive living with PD. In another qualitative study, people with PD perceived family support as important for positive adjustment to the disease. Based on our findings, it seems that social distancing and the lockdown affected this aspect of life among only a small portion of the respondents. It is possible that the main source of social support for the respondents in this study was their close family with whom they live, and that they were able to maintain connections with friends via phone or other media such as video calls.

Among the respondents, 85.8% used rehabilitative care services in the period before the pandemic. This enabled us to ascertain the extent of disruption in this aspect of care. Rehabilitative care is an important component of healthcare among people with PD [41]. It is also a primary factor supporting the function and well-being of people with PD, as indicated by clinical guidelines [42]. It seems that while measures were taken to ensure that people are able to maintain medical and pharmacological care, such as offering telemedicine or the home delivery of prescribed drugs, less attention was given to maintaining rehabilitative care. In addition, it is likely that the frequency and delivery methods of rehabilitative care made this aspect of care more difficult to implement while maintaining social distancing. The alternatives to in-person treatments provided by healthcare organizations, such as remote therapy guided by health professionals, did not effectively replace routine rehabilitative therapy and community exercise programs.

Our findings suggest that healthcare systems should strategize how to effectively deliver rehabilitative care, even when social distancing is being enforced. Moreover, healthcare professions should integrate intervention strategies that empower patients, such as self-management support and techniques for treating patients remotely, into their curricula.

#### Patient activation and its association with changes in health and well-being

This is the first study to present measures of patient activation in people with PD, and more importantly, to demonstrate the significant role of activation in the ability to maintain function and health during a major disruption in life routines.

According to our hypothesis, among respondents with higher levels of activation, social distancing and the lockdown had a lesser effect on the worsening of disease symptoms, increased tiredness, and lack of support from family members and friends. The strengths of these associations ranged from fair to weak. Furthermore, the amount of change in these domains differed between respondents with high and low levels of activation. Studies involving other health conditions have reported associations similar to those found in our study [25, 43–45]. Overall, these findings suggest that greater perceived patient engagement in managing their health condition results in a range of improved health and well-being outcomes.

#### Study limitations

The study findings may not be generalizable to all people with PD as the respondents in this study were well supported by their spouses, engaged regularly in physical activity or rehabilitative care, and were computer or smartphone literate. We speculate that underprivileged people, whom we were unable to include in the study, may have experienced stronger negative effects of social distancing and the lockdown. In addition, this study lacked information about the respondents’ level of physical activity before the COVID-19 induced social distancing; this prevented us from assessing changes in physical activity levels.

### Conclusion

Although the COVID-19 pandemic may be a temporary crisis, its effects could have long-term consequences. Therefore, we suggest that the healthcare community adopt strategies to minimize the negative effects of future similar crises. Strategies should be developed to maintain the delivery of rehabilitative care and exercise even during social distancing, since a lack of these was perceived by the respondents as an important contributor to the deterioration of their health. A link between the patients’ knowledge, skills, and confidence in managing their health and negative effects of social distancing may indicate that supporting these aspects will help people maintain their physical and mental health in times of dramatic changes in life’s circumstances.

### Practice implications

Our findings highlight the importance of fostering patient’s activation as a tool to improve their function, health, and well-being [25]. The findings extend current knowledge by demonstrating that “activation” empowers people not only in their daily life, but also when they experience stressful and limiting circumstances. The concept of patient activation is located within the broader context of self-management. Therefore, our findings strengthen the call to embed self-management support as an integral part of health treatment in people with PD. Under the current scenario, telehealth, which offers remote consultations with health professionals and exercise, may be an important avenue to support self-management [46]. The COVID-19 pandemic is expected to facilitate the development of telehealth services and its implementation, specifically for people with PD [7, 8, 12, 47]. Self-management support may also involve education about the disease [48] and community self-management groups that focus on the specific skills needed for effective self-management, such as problem solving and resource utilization [49].

## Supplementary Information

**Additional file 1: Appendix 1.** Complete survey results.

## Data Availability

All data generated or analysed during this study are included in this published article (see Appendix 1). For information about the data, please contact Galit Yogev-Seligmann email: galit.yogev@gmail.com.

## References

[1] Dahodwala N, Willis AW, Li P, Doshi J (2017). Prevalence and correlates of anti-Parkinson drug use in a nationally representative sample. Mov Disord Clin Pract.

[2] Willis AW, Kung N, Perlmutter JS, Racette BA (2012). Neurologist-associated reduction in PD-related hospitalizations and health care expenditures. Neurology.

[3] Willis W (2011). Neurologist care in Parkinson disease A utilization, outcomes, and survival study. Neurology.

[4] Ney JP, Johnson B, Knabel T, Craft K (2016). Neurologist ambulatory care, health care utilization, and costs in a large commercial dataset. Neurology.

[5] Wei YJ, Palumbo FB, Simoni-Wastila L, Shulman LM, Stuart B, Beardsley R (2015). Relationships between antiparkinson medication nonadherence, regimen modifications, and healthcare utilization and expenditures. Park Relat Disord.

[6] Ambrosio L, Portillo MC, Rodriguez-Blazquez C, Rojo JM, Martinez-Martin P, EC-PC Validation Group, Violante MR, Castrillo JC, Arillo VC, Garretto NS, Arakaki T. Influencing factors when living with Parkinson’s disease: A cross-sectional study. J Clin Nurs. 2019;28(17-18):3168–76. 10.1111/jocn.14868 30938889

[7] Papa SM, Brundin P, Fung VSC, Kang UJ, Burn DJ, Colosimo C (2020). Impact of the COVID-19 Pandemic on Parkinson’s disease and movement disorders. Mov Disord.

[8] Bhaskar S, Bradley S, Israeli-Korn S, Menon B, Chattu VK, Thomas P, et al. Chronic neurology in COVID-19 era: clinical considerations and recommendations from the REPROGRAM consortium. Front Neurol. 2020;11:664. 10.3389/fneur.2020.00664 PMC7339863 32695066

[9] Alessi J, De Oliveira GB, Franco DW, Brino Do Amaral B, Becker AS, Knijnik CP, et al. Mental health in the era of COVID-19: prevalence of psychiatric disorders in a cohort of patients with type 1 and type 2 diabetes during the social distancing. Diabetol Metab Syndr. 2020;12(1):1–10. 10.1186/s13098-020-00584-6 PMC7457442 32879637

[10] Elbeddini A, To A, Tayefehchamani Y, Wen C. Potential impact and challenges associated with Parkinson’s disease patient care amidst the COVID-19 global pandemic. J Clin Mov Disord. 2020;7(1):1–7. 10.1186/s40734-020-00089-4 PMC7414276 32782815

[11] Bhidayasiri R, Virameteekul S, Kim JM, Pal PK, Chung SJ. COVID-19: An early review of its global impact and considerations for parkinson’s disease patient care. J Mov Disord. 2020;13(2):105–14. 10.14802/jmd.20042 PMC7280938 32344993

[12] Helmich RC, Bloem BR. The impact of the COVID-19 pandemic on parkinson’s disease: hidden sorrows and emerging opportunities. J Park Dis. 2020;10(2):351. 10.3233/JPD-202038 PMC7242824 32250324

[13] Kumar, N, Gupta, R, Kumar, H, Mehta, S, Rajan, R, Kumar, D, et al. Impact of home confinement during COVID-19 pandemic on Parkinson’s disease. Park Relat Disord. 2021;77:15–22. 10.1016/j.parkreldis.2020.09.003 PMC7474806 32937224

[14] Schirinzi T, Di Lazzaro G, Salimei C, Cerroni R, Liguori C, Scalise S, et al. Physical activity changes and correlate effects in patients with parkinson’s disease during COVID-19 Lockdown. Mov Disord Clin Pract. 2020;7(7):797–802. 10.1002/mdc3.13026 PMC7404747 32837960

[15] Song J, Ahn JH, Choi I, Mun JK, Cho JW, Youn J. The changes of exercise pattern and clinical symptoms in patients with Parkinson’s disease in the era of COVID-19 pandemic. Park Relat Disord. 2020;80:148-51. 10.1016/j.parkreldis.2020.09.034 PMC7510770 33002722

[16] Guo D, Han B, Lu Y, Lv C, Fang X, Zhang Z, et al. Influence of the COVID-19 pandemic on quality of life of patients with parkinson’s disease. Park Dis. 2020. 10.1155/2020/1216568. 10.1155/2020/1216568 PMC7537675 33062247

[17] Del Prete E, Francesconi A, Palermo G, Mazzucchi S, Frosini D, Morganti R, et al. Prevalence and impact of COVID-19 in Parkinson’s disease: evidence from a multi-center survey in Tuscany region. J Neurol. 2021;268(4):1179–87. 10.1007/s00415-020-10002-6 PMC7471534 32880722

[18] De Micco R, Siciliano M, Sant’Elia V, Giordano A, Russo A, Tedeschi G, et al. A. Correlates of psychological distress in Parkinson’s disease patients during the COVID‐19 outbreak. Mov Disord Clin Pract. 2020;8:60–8. 10.1002/mdc3.13108 PMC7780948 33426160

[19] Grady PA, Gough LL (2018). Self-management: a comprehensive approach to management of chronic conditions. Am J Public Health.

[20] Nolte E, Knai C, Saltman RB, eds. Assessing chronic disease management in European health systems: Concepts and approaches. Copenhagen (Denmark): European Observatory on Health Systems and Policies; 2014. 29019637

[21] Brady TJ, Sacks JJ, Terrillion AJ, Colligan EM (2018). Operationalizing surveillance of chronic disease self-management and self-management support. Prev Chronic Dis.

[22] Samson A, Siam H (2008). Adapting to major chronic illness: a proposal for a comprehensive task-model approach. Patient Educ Couns.

[23] Hibbard JH, Mahoney ER, Stock R, Tusler M (2007). Self-management and health care utilization. Health Serv Res.

[24] Greene J, Hibbard JH. Why does patient activation matter ? An examination of the relationships between patient activation and health-related outcomes. J Gen Intern Med. 2012;27(5):520–6. 10.1007/s11606-011-1931-2 PMC3326094 22127797

[25] Greene J, Hibbard JH, Sacks R, Overton V, Parrotta CD (2015). When patient activation levels change, health outcomes and costs change, too. Health Aff.

[26] Mosen DM, Schmittdiel J, Hibbard J, Sobel D, Remmers C, Bellows J (2007). Is patient activation associated with outcomes of care for adults with chronic conditions?. J Ambul Care Manage.

[27] Hibbard JH, Stockard J, Mahoney ER, Tusler M (2004). Development of the Patient Activation Measure (PAM): conceptualizing and measuring activation in patients and consumers. Health Serv Res.

[28] Hibbard JH, Mahoney ER, Stockard J, Tusler M. Development and testing of a short form of the patient activation measure. Health Serv Res. 2005;40(6 Pt 1):1918–30. 10.1111/j.1475-6773.2005.00438.x PMC1361231 16336556

[29] Packer TL, Kephart G, Ghahari S, Audulv Å, Versnel J, Warner G (2015). The patient activation measure: a validation study in a neurological population. Qual Life Res.

[30] Chan YH (2003). Biostatistics 104: correlational analysis. Singapore Med J.

[31] Oppo V, Serra G, Fenu G, Murgia D, Ricciardi L, Melis M, et al. Parkinson’s disease symptoms have a distinct impact on caregivers’ and patients’ stress: a study assessing the consequences of the COVID-19 lockdown. Mov Disord Clin Pract. 2020;7(7):865–7. 10.1002/mdc3.13030 PMC7533970 33043088

[32] Okuma Y, de Lima ALS, Fukae J, Bloem BR, Snijders AHA (2018). prospective study of falls in relation to freezing of gait and response fluctuations in Parkinson’s disease. Parkinsonism Relat Disord.

[33] Gazibara T, Pekmezovic T, Tepavcevic DK, Tomic A, Stankovic I, Kostic VS, Svetel M (2014). Circumstances of falls and fall-related injuries among patients with Parkinson’s disease in an outpatient setting. Geriatr Nurs.

[34] Lamont RM, Morris ME, Menz HB, McGinley JL, Brauer SG (2017). Falls in people with Parkinson’s disease: a prospective comparison of community and home-based falls. Gait Posture.

[35] Marsh L (2013). Depression and Parkinson’s disease: current knowledge. Curr Neurol Neurosci Rep.

[36] Schrag A. Quality of life and depression in Parkinson’s disease. J Neurol Sci. 2006;248(1-2):151–7. 10.1016/j.jns.2006.05.030 16797028

[37] Holroyd S, Currie LJ, Wooten GF. Depression is associated with impairment of ADL, not motor function in Parkinson disease. Neurology. 2005;64(12):2134–5. 10.1212/01.WNL.0000165958.12724.0D 15985588

[38] Wiesli D, Meyer A, Fuhr P, Gschwandtner U. Influence of mild cognitive impairment, depression, and anxiety on the quality of life of patients with Parkinson disease. Dement Geriatr Cogn Dis Extra. 2017;7(3):297–308. 10.1159/000478849 PMC5662998 29118782

[39] Lerdal A, Lee KA, Bakken LN, Finset A, Kim HS. The course of fatigue during the first 18 months after first-ever stroke: a longitudinal study. 2012;2012:126275. 10.1155/2012/126275 PMC3189618 22007350

[40] Friedman JH, Brown RG, Comella C, Garber CE, Krupp LB, Lou JS, et al. Fatigue in Parkinson’s disease: a review. Mov Disord. 2007;22(3):297–308. 10.1002/mds.21240 17133511

[41] Bloem BR, de Vries NM, Ebersbach G (2015). Nonpharmacological treatments for patients with Parkinson’s disease. Mov Disord.

[42] Keus HSJ, Munneke M, Graziano M, Paltamaa J, pelosin E, Domingos J, et al. European physiotherapy guidline for parkinson’s disease. The Netherlands: KNGF/ParkinsonNet; 2014.

[43] Kukla M, Salyers MP, Lysaker PH (2013). Levels of patient activation among adults with schizophrenia: associations with hope, symptoms, medication adherence, and recovery attitudes. J Nerv Ment Dis.

[44] Smith SG, Curtis LM, Wardle J, von Wagner C, Wolf MS (2013). Skill set or mind set ? Associations between Health Literacy, patient activation and health. PLoS One.

[45] Magnezi R, Glasser S, Shalev H, Sheiber A, Reuveni H (2014). Patient education and counseling patient activation, depression and quality of life. Patient Educ Couns.

[46] Guo Y, Albright D. The effectiveness of telehealth on self-management for older adults with a chronic condition: a comprehensive narrative review of the literature. J Telemed Telecare. 2018;24(6):392–40. 10.1177/1357633X17706285 28449619

[47] Subramanian I. Virtual Parkinson’s Disease Support Groups in the COVID-19 Era: social connection in the time of social distancing. Mov Disord Clin Pract. 2020;7(6):739–40. 10.1002/mdc3.12994 PMC7300459 32775536

[48] Kessler D, Liddy C (2017). Self-management support programs for persons with Parkinson’s disease: an integrative review. Patient Educ Couns.

[49] Nelson N, Wong D, Lai E. A self-management program for veterans and spouses living with Parkinson’s disease. J Nurs Healthc Chronic Illn. 2011;3(4):496–503.

