# Supplementary material for: COVID-19 social distancing: negative effects on people with Parkinson disease and their associations with confidence for self-management
Source: BMC Neurol. 2021 Jul 20;21:284. doi: 10.1186/s12883-021-02313-6 (PMC8289714; doi:10.1186/s12883-021-02313-6)
Supplement: Supplementary file 1 — Additional file 1: Appendix 1. Complete survey results. [file 12883_2021_2313_MOESM1_ESM.docx]

COVID-19 Social Distancing: Negative Effects on People with Parkinson Disease and their associations with confidence for Self-Management

Galit Yogev-Seligmann, BPT, PhD, Michal Kafri, BPT, PhD

Appendix: **Complete survey results** (results are presented as percentage (%) of respondents)

| **Q1. Do you use a walking aid?** | | | | | | | | | | | | | | | | | | | | | | | | | | | | | |
| --- | --- | --- | --- | --- | --- | --- | --- | --- | --- | --- | --- | --- | --- | --- | --- | --- | --- | --- | --- | --- | --- | --- | --- | --- | --- | --- | --- | --- | --- |
| I walk without a walking aid | | | | I walk with a walking cane | | | | | | | | | | I walk with a walker | | | | | | | | | I get around in a wheelchair | | | | | | |
| 60.2% | | | | 17.9% | | | | | | | | | | 15.8% | | | | | | | | | 6.1% | | | | | | |
| **Q2. Do you require help from another person for walking?** | | | | | | | | | | | | | | | | | | | | | | | | | | | | | |
| I walk independently | | | | | | | | During walking, I require another person's supervision | | | | | | | | | | | | During walking, I require another person's physical support and help | | | | | | | | | |
| 68.2% | | | | | | | | 21.4% | | | | | | | | | | | | 10.4% | | | | | | | | | |
| **Q3. Compared to the period before the COVID-19 pandemic, has there been any change in your ability to walk?** | | | | | | | | | | | | | | | | | | | | | | | | | | | | | |
| No, my walking ability has not changed | | | | | | | | Yes, my walking ability has changed for the worse | | | | | | | | | | | | Yes, my walking ability has improved | | | | | | | | | |
| 62.6% | | | | | | | | 35.9% | | | | | | | | | | | | 1.5% | | | | | | | | | |
| **Q4. Do you require help from another person in basic daily functions (clothing, bathing, eating)?** | | | | | | | | | | | | | | | | | | | | | | | | | | | | | |
| I am independent in all my daily functions | | | | I require supervision in some or all of my daily functions | | | | | | | | | | I require a little help in some or all of my daily functions | | | | | | | | | I require medium to great help in some or all of my daily functions | | | | | | |
| 54.0% | | | | 12.6% | | | | | | | | | | 21.2% | | | | | | | | | 12.1% | | | | | | |
| **Q5.** **Compared to the period before the COVID-19 pandemic, has there been any change in the amount of help that you currently require?** | | | | | | | | | | | | | | | | | | | | | | | | | | | | | |
| No, I function like before the COVID-19 pandemic | | | | | | | | Yes, I require more help | | | | | | | | | | | | Yes, I require less help | | | | | | | | | |
| 74.6% | | | | | | | | 25.4% | | | | | | | | | | | | - | | | | | | | | | |
| **Q6. Did you feel depressed during the COVID-19 period?** | | | | | | | | | | | | | | | | | | | | | | | | | | | | | |
| Not at all | | A little | | | | | | | | | | Sometimes | | | | | | Often | | | | | | | | | All the time | | |
| 37.1% | | 22.8% | | | | | | | | | | 26.4% | | | | | | 12.7% | | | | | | | | | 1.0% | | |
| **Q7. Did you feel isolated and lonely during the COVID-19 period?** | | | | | | | | | | | | | | | | | | | | | | | | | | | | | |
| Not at all | | A little | | | | | | | | | | Sometimes | | | | | | Often | | | | | | | | | All the time | | |
| 45.2% | | 23.4% | | | | | | | | | | 19.3% | | | | | | 9.6% | | | | | | | | | 2.5% | | |
| **Q8. Did you feel anxious during the COVID-19 period?** | | | | | | | | | | | | | | | | | | | | | | | | | | | | | |
| Not at all | | A little | | | | | | | | | | Sometimes | | | | | | Often | | | | | | | | | All the time | | |
| 44.7% | | 27.9% | | | | | | | | | | 19.3% | | | | | | 6.6% | | | | | | | | | 1.5% | | |
| **Q9. Did you feel worried about your future during the COVID-19 period?** | | | | | | | | | | | | | | | | | | | | | | | | | | | | | |
| Not at all | | A little | | | | | | | | | | Sometimes | | | | | | Often | | | | | | | | | All the time | | |
| 34.4% | | 32.8% | | | | | | | | | | 21.5% | | | | | | 9.2% | | | | | | | | | 2.1% | | |
| **Q10. In the previous three questions, you were asked about feelings of depression, loneliness, anxiety and concern for the future. Has one or more of these feelings changed during the COVID-19 period compared to the period before it?** | | | | | | | | | | | | | | | | | | | | | | | | | | | | | |
| No, I felt the same before the COVID-19 pandemic | | | | | | | | | Yes, before the COVID-19 pandemic I felt better | | | | | | | | | | | | Yes, before the COVID-19 pandemic I felt worse | | | | | | | | |
| 55.7% | | | | | | | | | 43.3% | | | | | | | | | | | | 1.0% | | | | | | | | |
| **Q11. Have you lacked the support you needed from your spouse during the COVID-19 period?** | | | | | | | | | | | | | | | | | | | | | | | | | | | | | |
| Not at all | A little | | | | | | | | Sometimes | | | | | | Often | | | | | | All the time | | | | | | | | I don’t have a spouse |
| 62.4% | 10.7% | | | | | | | | 9.1% | | | | | | 4.1% | | | | | | 1.5% | | | | | | | | 12.2% |
| **Q12. Have you lacked the support you needed from your family members or your close friends during the COVID-19 period?** | | | | | | | | | | | | | | | | | | | | | | | | | | | | | |
| Not at all | | A little | | | | | | | | | | Sometimes | | | | | | Often | | | | | | | | | All the time | | |
| 51.3% | | 23.4% | | | | | | | | | | 14.7% | | | | | | 7.1% | | | | | | | | | 3.6% | | |
| **Q13. Have you fallen during the COVID-19 period?** | | | | | | | | | | | | | | | | | | | | | | | | | | | | | |
| No | | | | | | | | | | | | | | | Yes | | | | | | | | | | | | | | |
| 81.7% | | | | | | | | | | | | | | | 18.3% | | | | | | | | | | | | | | |
| **Q14. Have you fallen more during the COVID-19 period compared to before this period?** | | | | | | | | | | | | | | | | | | | | | | | | | | | | | |
| No | | | | | | | | | | | | | | | Yes | | | | | | | | | | | | | | |
| 95.4% | | | | | | | | | | | | | | | 4.6% | | | | | | | | | | | | | | |
| **Q15. If you suffer from other comorbidities, such as hypertension, diabetes or heart disease, have you controlled these comorbidities during the COVID-19 period?** | | | | | | | | | | | | | | | | | | | | | | | | | | | | | |
| Yes | | | | | | | | | | | | | | | No | | | | | | | | | | | | | | |
| 85.6% | | | | | | | | | | | | | | | 14.4% | | | | | | | | | | | | | | |
| **Q16. How much during the day have you felt tired during the COVID-19 period?** | | | | | | | | | | | | | | | | | | | | | | | | | | | | | |
| I did not feel tired at all | | | | | I felt tired in a small part of the day | | | | | | | | | | I felt tired in part of the day | | | | | | | | | I felt tired most of the day | | | | | |
| 11.3% | | | | | 34.4% | | | | | | | | | | 41.0% | | | | | | | | | 13.3% | | | | | |
| **Q17. Compared to the period before the COVID-19 pandemic, has there been any change in the degree of your tiredness during the COVID-19 period?** | | | | | | | | | | | | | | | | | | | | | | | | | | | | | |
| My tiredness has not changed | | | | | | | | | | My tiredness has increased | | | | | | | | | | | My tiredness has decreased | | | | | | | | |
| 55.3% | | | | | | | | | | 42.6% | | | | | | | | | | | 2.0% | | | | | | | | |
| **Q18. Compared to the period before the COVID-19, have there been any changes in your body weight during the COVID-19 period?** | | | | | | | | | | | | | | | | | | | | | | | | | | | | | |
| My body weight has not changed | | | | | | | | | | I have gained weight | | | | | | | | | | | I have lost weight | | | | | | | | |
| 59.9% | | | | | | | | | | 29.9% | | | | | | | | | | | 10.2% | | | | | | | | |
| **Q19. If you receive any rehabilitative therapy (physical therapy, occupational therapy, speech therapy), has the COVID-19 pandemic affected your ability to receive these treatments?** | | | | | | | | | | | | | | | | | | | | | | | | | | | | | |
| Yes, my treatments have been discontinued by the service provider | | | | | | Yes, I was afraid and I did not want to continue the treatments I was used to | | | | | | | | | | No, I received my treatments as usual | | | | | | | | | I did not receive any rehabilitation treatments before or during the COVID-19 period | | | | |
| 57.8% | | | | | | 10.3% | | | | | | | | | | 16.8% | | | | | | | | | 15.1% | | | | |
| **Q20. Has there been a change in the symptoms related to your disease during the COVID-19 period? If you suffer of Parkinson’s disease, symptoms may include: Freezing of gait, rigidity, postural imbalance, tremor. If you are a person who had a stroke, symptoms may include increase in muscle tone, worsening of limb weakness, lack of balance and pain.** | | | | | | | | | | | | | | | | | | | | | | | | | | | | | |
| The symptoms of my disease have not changed | | | | | | | | | | My symptoms have worsened | | | | | | | | | | | My symptoms have improved | | | | | | | | |
| 57.4% | | | | | | | | | | 41.1% | | | | | | | | | | | 1.5% | | | | | | | | |
| **Q21. If the condition of your disease worsened during the COVID-19 period, which of the following do you think explains the worsening?** | | | | | | | | | | | | | | | | | | | | | | | | | | | | | |
| Lack of contact with friends and family | | | Inability to visit a neurologist | | | | | | | | | | Lack of exercise or physical activity I have done before | | | | | | Lack of rehabilitation therapies (physical therapy, occupational therapy or speech therapy) | | | | | | | | | Other reasons | |
| 13.3% | | | 5.5% | | | | | | | | | | 36.7% | | | | | | 32.0% | | | | | | | | | 12.5% | |
| **Q22.** **How often do you perform physical exercise?** | | | | | | | | | | | | | | | | | | | | | | | | | | | | | |
| I exercise every day | | | | | | I exercise 3-5 times a week | | | | | | | | | | I exercise 1-2 times a week | | | | | | | | | I don't exercise | | | | |
| 31.3% | | | | | | 36.9% | | | | | | | | | | 18.7% | | | | | | | | | 13.1% | | | | |
| **Q23. During the COVID-19 period, what kind of exercise did you perform?** [open ended question] | | | | | | | | | | | | | | | | | | | | | | | | | | | | | |
| **Q24.** **Have you kept taking medication for your disease according to your doctor's instructions during the COVID-19 period?** | | | | | | | | | | | | | | | | | | | | | | | | | | | | | |
| Yes | | | | | | | | | | Sometimes | | | | | | | | | | | No | | | | | | | | |
| 97.0% | | | | | | | | | | 2.5% | | | | | | | | | | | 0.5% | | | | | | | | |
| Q25. **Compared to the period before the COVID-19 pandemic, has there been any change in your medication?** | | | | | | | | | | | | | | | | | | | | | | | | | | | | | |
| Yes, since I could not buy the medication I need | | | Yes, since I could not get to the hospital in order to receive the medical treatment I need | | | | | | | | | | Yes, since I forgot to take my medication more than usual | | | | | | Yes, since I could not buy the medication I need | | | | | | | | | There has been no change | |
| 2.6% | | | 0.5% | | | | | | | | | | 2.0% | | | | | | 5.1% | | | | | | | | | 89.8% | |
| Q26. **Have you been in touch with your neurologist during the COVID-19 period?** | | | | | | | | | | | | | | | | | | | | | | | | | | | | | |
| Yes, I have been in touch with my neurologist, like before the COVID-19 pandemic | | | | | | | Yes, I have been in touch with my neurologist more than before the COVID-19 pandemic | | | | | | | | | | Yes, but I was less in touch with my neurologist due to the COVID-19 pandemic | | | | | | | | | No, I have not been in touch with my neurologist, but this has nothing to do with the COVID-19 pandemic | | | |
| 25.9% | | | | | | | 2.0% | | | | | | | | | | 7.6% | | | | | | | | | 64.5% | | | |
| Q27. **The condition of your disease now…** | | | | | | | | | | | | | | | | | | | | | | | | | | | | | |
| Has not changed compared to the period before the COVID-19 pandemic | | | | | | | | | | | Is better compared to the period before the COVID-19 pandemic | | | | | | | | | | | Is worse compared to the period before the COVID-19 | | | | | | | |
| 66.2% | | | | | | | | | | | 2.0% | | | | | | | | | | | 31.8% | | | | | | | |
